# A cross-sectional study on the reasons for vaccine hesitancy in children under seven years of age in Isfahan, Iran

**DOI:** 10.1016/j.jvacx.2023.100396

**Published:** 2023-10-11

**Authors:** Negar Farajzadeh, Habibollah Hosseini, Mahrokh Keshvari, Mohammad R. Maracy

**Affiliations:** a Master Student of Community Health Nursing, School of Nursing and Midwifery, Isfahan University of Medical Sciences, Isfahan, Iran; b Nursing and Midwifery Care Research Center, Faculty of Nursing and Midwifery, Isfahan University of Medical Sciences, Isfahan, Iran; c Department of Epidemiology & Biostatistics, School of Public Health, Isfahan University of Medical Sciences, Isfahan, Iran

**Keywords:** Vaccination, Vaccine hesitancy, Vaccines, Child, Immunization

## Abstract

**Introduction:**

The diminution in vaccination coverage in recent years has contributed to the reappearance of infectious diseases, and vaccine hesitancy is one of the main causes. As a result, we investigated the causes of vaccine hesitancy in children.

**Materials and methods:**

This descriptive-analytical study was conducted cross-sectionally in 27 comprehensive health service centers in Isfahan City from June to October 2022. This study included Iranian families living in Isfahan who did not vaccinate their children by the due date. A researcher-made questionnaire collected data on children's vaccine hesitancy after verifying validity and reliability over the phone and in person by the researcher. The mothers completed informed consent. Independent T-tests, Pearson's correlation coefficient, analysis of variance, and a generalized linear model were used to analyze the data.

**Findings:**

Finally, 298 families participated in the study, 34.3% refused, and 65.7% delayed their child's vaccination. Vaccination was hesitant due to the child being sick at the time of injection (57.3%), believing that vaccination is not necessary to prevent uncommon diseases (49%), and being concerned about severe side effects caused by the vaccine (48.7%). Vaccine hesitancy among mothers with bachelor's degrees and families less than one kilometer from the health center was significantly less than among others. Additionally, Vaccines at birth, four, six, twelve, and eighteen months, and six-year-old vaccines were associated with vaccine hesitancy.

**Conclusion:**

Children's illness at the time of vaccination, the belief that vaccination is unnecessary to prevent uncommon diseases, and the worry about severe complications were among the most common reasons for vaccine hesitancy. Mother's education, distance to the health center, and vaccine type were associated with vaccine hesitancy.

## Introduction

Vaccination is one of the most successful public health interventions in preventing the death of 2–3 million children annually [1]. However, unfortunately, its coverage has decreased significantly in the last decade. A growing number of parents in the industrialized world prefer not to vaccinate their children [2]. For example, in 2021, about 25 million children will not have been vaccinated, This is 5.9 million more than in 2019, the highest number since 2009 [3]. From 2019 to 2021, global vaccination coverage decreased from 86% to 81% [4], [5]. Decreased coverage has historically been thought to largely result from barriers to vaccine access [6]. But recently, vaccine hesitancy has been recognized as an important emerging risk factor for non-vaccination [7].

The World Health Organization considers vaccine hesitancy to include delay in accepting or refusing vaccination despite access to vaccination services [8]. Vaccine hesitancy has been a phenomenon since vaccine introduction [9]. However, conflicting information and evidence-based evidence have supported and reinforced it in recent decades. Vaccine hesitancy has many reasons and is affected by contextual, individual, group, and vaccine-specific issues [10].

WHO^1^ examined three main reasons for vaccine hesitancy in 17 countries. These reasons included religious beliefs, fear of vaccine side effects, receiving negative information from the media, and mistrusting and seeing vaccines as useless [11]. Several studies also stated that the reasons for vaccine hesitancy are related to insufficient knowledge about the importance of vaccines; They also noted a lack of knowledge about the immunization schedule and the child being sick at the time of injection [12]. Demographic factors such as the age and gender of the child, the number of children in the family and their birth rank, education and occupation of the parents, place of residence, and distance to the vaccination center can be mentioned among the other most influential factors in vaccination [13], [14], [15], [16], [17]. Vaccine hesitancy depends on several factors and needs to be studied in the same environment. Unfortunately, vaccine hesitancy has increased in Iran [18].

According to Iran's Ministry of Health report, vaccination coverage has dropped below 95% [19]. In 2022 in Isfahan, we saw the reappearance of some diseases, such as measles [20]. Considering the importance and necessity of high vaccination coverage, knowing more about the causes of vaccine hesitancy is necessary. This is to plan to solve these issues and reduce hesitation. This study aimed to determine the reasons of vaccine hesitancy of children under seven in families covered by comprehensive health service centers in Isfahan.

## Materials and methods

### Research environment and people

This descriptive-analytical study was conducted cross-sectionally in Isfahan on families covered by comprehensive health service centers from June to October 2022. Isfahan City has two health centers. Each center includes several comprehensive urban and rural health service centers. Each center was geographically divided into five regions to select urban centers. Two centers were randomly selected from each area. Urban and rural centers were also chosen randomly in each center separately. A total of 20 urban centers, five suburban centers, and two rural centers were selected. The research subjects were families with at least one child under seven (6 years, 11 months, and 29 days) who were not vaccinated on time.

Other inclusion criteria included a delay of more than one day or refusal of vaccination, having Iranian nationality, living in Isfahan city, guardianship of the child by the father or mother, and the child being alive during the research. If a family did not want to complete the questionnaire or gave the same answers to all the questions, it was excluded from the study despite the reverse questions.

### Tools

Data collection was done using a researcher-made questionnaire about children's vaccinations. The first part of the questionnaire included demographic information. The second part was taken from the standard questions developed by the WHO Strategic Advisory Group of Experts (SAGE) on Immunization [11]. Twenty experts measured quantitative and qualitative face and content validity. After calculating the CVI^2^and CVR^3^of each question, necessary changes were made to the questionnaire.

The external reliability was tested by 40 families who met the study entry criteria by the test–retest method twice with an interval of 14 days. The Pearson correlation coefficient was 0.98, and Cronbach's alpha coefficient was 0.71, indicating internal and external satisfaction reliability. It was part of a questionnaire. This questionnaire had 30 questions on a Likert scale. The strongly agree, agree, have no opinion, disagree, and strongly disagree options were given 5 to 1, respectively. Seven negative statements were also scored inversely. The minimum total score of the questionnaire was 58, and the maximum was 122.

### Methods

After extracting the total number of children under seven years of age with delayed vaccination from the SIB system (an integrated health system for registering, maintaining, and updating the electronic health record information of Iranians) and specifying the centers using the ratio distribution method, the required sample size from each center was achieved.

Based on the SIB list, the researcher visited the selected centers, selected people by systematic sampling, and contacted their mothers. Having explained the research objectives to mothers, they were invited to attend the center. The researcher personally asked the mothers the questionnaire questions after they completed the informed consent form. When the mother could not attend the centers or refused to attend, the researcher conducted telephone interviews with them. Completing each questionnaire took 10 to 15 min.

### Data analysis

In this research, for the primary analysis of the child's gender variable from the independent *t*-test, the child's age and mother's age variables from the correlation test, and the variables of the child's birth rank in the family, the type of vaccine, the mother's education, mother's occupation, place of residence and distance to the health center from the test Analysis of variance was used. The final analysis was conducted by entering all variables into a generalized linear model (GLM) and measuring their effects. Data analysis was done using SPSS version 20. A significance level of 0.5 was considered the significance limit, and a 95% confidence interval was used to evaluate the results' accuracy.

## Results

**Fig. 1:**
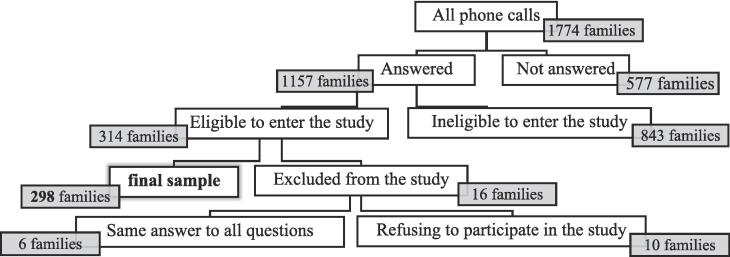
The process of selecting research samples.

In this study, 1774 families were contacted by phone, 1157 (67%) responded, and finally, only 298 (17%) met the inclusion criteria and participated in the study. Fig. 1 illustrates the process of selecting research samples.

In this study, the children were 2 to 83 months. Most (51.3%) were daughters and the second child (38.6%) The mothers' ages ranged from 20 to 46 years, and most of them (79.9%) were housewives and had bachelor's degrees (42.3%). Most families live in urban areas less than one kilometer from the health center. There was the most hesitancy about 18-month vaccines and the slightest hesitancy about birth vaccines.

The average vaccine hesitancy according to demographic factors is shown in Table 1. Mothers with a bachelor's degree obtained a lower vaccine hesitancy score than other mothers. The generalized linear model showed this difference to be significant. Also, in the generalized linear model, a significant relationship was found between vaccine hesitancy and distance to health centers. Families with a distance of less than one kilometer to the health center had less hesitancy to receive vaccines than those with more than one kilometer.

**Table 1:** The average score of vaccine hesitancy by demographic factors.

Variables	Characteristics	N = 298 n (%)	Mean ± SD	P.Crude	P.Value Adjust*
Child's gender	Girl	153 (51.3)	75.2 ± 13.6	0.231	0.508
	Boy	145 (48.7)	77.3 ± 13.8		
Child's age	Month(s)	298 (100)	–	0.880	0.120
Child's birth rank	First	114 (38.3)	74.7 ± 12.8	0.215	0.368
	Second	114 (38.6)	76.1 ± 13.9		
	Third	54 (18.1)	79.5 ± 14.7		
	>3	15 (5)	76.3 ± 15.5		
Delayed vaccine type	Birth	10 (3.4)	74.8 ± 11.1	0.740	0.019
	2 months	32 (10.7)	77.9 ± 10.3	0.457	0.833
	4 months	58 (19.5)	78.1 ± 12.4	0.266	<0.001
	6 months	73 (24.5)	78.4 ± 12.4	0.116	<0.001
	12 months	109 (36.6)	78.7 ± 15.2	0.017	<0.001
	18 months	130 (43.6)	77.3 ± 12.4	0.204	<0.001
	6 years	35 (11.7)	72.9 ± 15.9	0.130	0.011
Mother's education	High school	31 (10.4)	74.8 ± 12.8	0.560	0.047
	diploma	76 (25.5)	76.9 ± 13.0		
	Associate Degree	32 (10.7)	73.6 ± 17.3		
	Masters	126 (42.3)	71.2 ± 12.4		
	Master's degree and above	33 (11.1)	74.4 ± 17.1		
Mother's job	housewife	238 (79.9)	76.0 ± 13.7	0.664	0.098
	non-householder	60 (20.1)	76.9 ± 13.8		
Mother's age	Year(s)	298 (100)	–	0.634	0.884
Place of residence	urban	243 (81.5)	75.6 ± 14.2	0.277	0.119
	suburban	44 (14.8)	78.6 ± 11.8		
	rural	11 (3.7)	79.9 ± 9.7		
Distance to the health center	<1 km	124 (41.6)	73.8 ± 14.1	0.152	0.024
	1–2 km	106 (35.6)	77.9 ± 13.0		
	2–3 km	39 (13.1)	77.8 ± 11.1		
	≥3 km	29 (7.9)	76.2 ± 13.7		

* Using the generalized linear model.

This study found a significant relationship between the vaccine hesitancy score and all childhood vaccines except the two-month vaccines. the two-month vaccines include Pentavalent (DTP + Hib + HepB) and OPV [21]. In other words, the vaccine hesitancy score of children who had doubts about the vaccines at birth, four, six, twelve, and eighteen months and six-year-old vaccines were significantly higher than children who did not have hesitancy about these vaccines. Furthermore, there was no significant difference in vaccine hesitancy scores between delayed and undelayed two-month vaccines children. There was no significant relationship between the vaccine hesitancy score and the age and gender of the child, the rank of the child, the mother's age and occupation, and the place of residence.

Among the studied families, the average vaccine hesitancy score was 76.22 ± 13.76, with a minimum of 34 and a maximum of 113. 34.3% of families refused to vaccinate their children and 65.7% delayed vaccination.

**Fig. 2:**
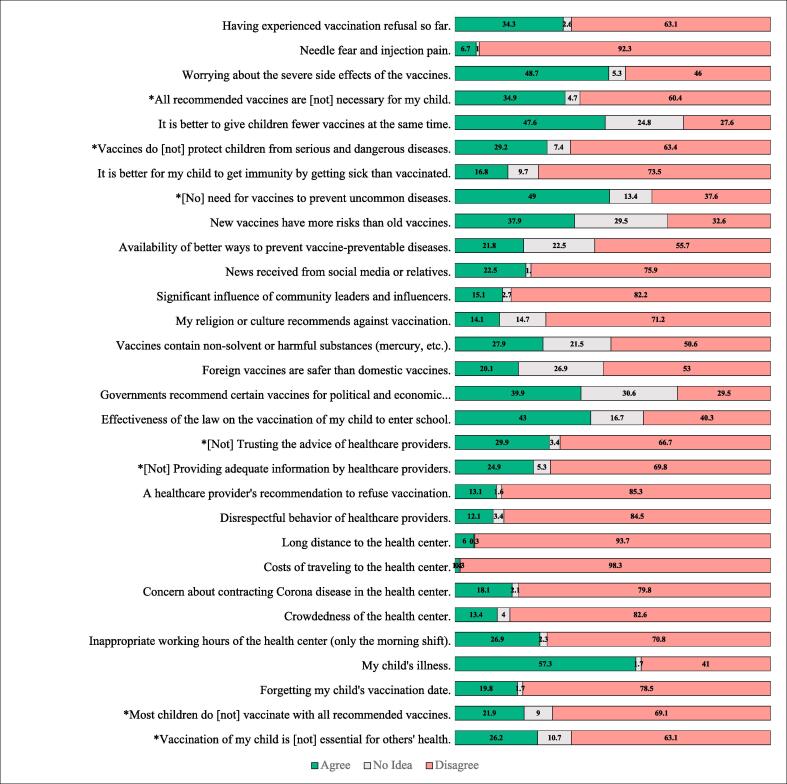
The reasons of vaccine hesitancy.

Fig. 2 shows the most common reasons for vaccine hesitancy of children in this study are the child being sick at the time of injection (57.3%), the belief that vaccination is not necessary for uncommon diseases (49%), and the fear of severe side effects (48.7%). There is a belief among 47.6% of mothers that children receive many vaccines each time. School enrollment was the only reason for vaccination for 43% of parents. According to 39.9% of mothers, governments recommend certain vaccines because of political and economic considerations. Most respondents (37.9% and 34.9%) agreed that new vaccines pose more risks than old vaccines and that all recommended vaccines are unnecessary.

## Discussion

This study aimed to determine the causes of vaccine hesitancy in children under seven years of age in Isfahan. In this study, no significant relationship was found between child age and vaccine hesitancy. This is consistent with studies conducted by Wagner et al. in India and China, Alsubaie et al. in Saudi Arabia, and Vasudevan et al. in Tanzania [13], [22], [23], [24]. Neither NikFarjam et al. in Tehran nor Domek et al. in Guatemala found a significant difference in vaccine hesitancy between boys and girls [16], [25]. Nevertheless, in the research conducted in Fars province by Mirahamdizadeh et al., girls were less hesitant about vaccination [14]. This difference can be due to the cultural difference between Fars and Isfahan in gender discrimination.

In the present study, unlike the research conducted in Iran by Rejali et al. (2015) and NikFarjam et al. (2016), no significant relationship was found between birth rate and vaccine hesitancy [1], [25]. This could be due to the decreased childbearing rates in recent years and the small number of third and older children in this study.

This study and other studies found no significant relationship between mothers' age and vaccine hesitancy. Unlike the present study and Luyten et al. (2019), Vasudevan et al. (2020), and Aslan et al. (2021) [13], [17], [26]; in Rejali et al. (2015), the mother's occupation had a significant relationship with vaccine hesitancy, and housewives had more hesitancy than working mothers [1]. This difference can be related to the type of mother's job; Therefore, evaluating the type of mother's job would be better in this study and other studies.

In this study, mothers' education had a significant relationship with vaccine hesitancy, but other studies reported different results. For instance, in the study of Luyten et al. in England and Melot et al. in Italy, no significant relationship was found between a mother's education and vaccine hesitancy [17], [27]. However, in studies conducted by Shapiro et al. in Canada, Wagner et al. in India, and Guay et al. in Quebec, the vaccine hesitancy rate decreased with the increase in mothers’ education [22], [28], [29]. Contrary, Alsubaie et al. in Saudi Arabia and Aslan et al. in Turkey showed the opposite, and with the increase in mothers' education, the vaccine hesitancy rate also increased [24], [26]. These differences can be attributed to the mother's field of study. For example, studying in areas related to religion or medical sciences can influence the factors that influence vaccine hesitancy. Therefore, evaluating mothers' educational backgrounds would be better in the present study and other studies.

There was no significant relationship between vaccine hesitancy and residence in the present study and studies conducted in Tehran, Guatemala, Tanzania, and Italy [13], [16], [25], [27]. Mirahmadizadeh et al. observed a significant difference between vaccine hesitancy in Fars’s rural and urban areas [14]. The difference with the present study is the proper and fair distribution of health care services in Isfahan City. In addition, the study conducts mobile visits to rural and urban centers. Isfahan's suburban and rural areas are also close to the urban areas in terms of distance and access, which may explain the lack of influence of place of residence on vaccine hesitancy. Unlike the studies conducted in India and Tanzania, this study found a significant relationship between distance and vaccine hesitancy [13], [22].

Unlike the two-month-old vaccines, this study revealed a significant relationship between vaccine hesitancy and vaccines at birth, four, six, twelve, eighteen months, and six-year-old vaccines. Most hesitancies were associated with 18-month vaccines, while minor hesitancies were associated with birth vaccines, which was consistent with studies by Rejali et al. (2015), Mirahmadizadeh et al. (2018), and NikFarjam et al. (2016) [1], [14], [25].

In the present study, the most common reasons for vaccine hesitancy are the child being sick at the time of vaccination, mothers' belief that vaccination is not needed for uncommon diseases, mothers' concern about severe complications caused by vaccines, and mothers' idea that the number of vaccines received in each turn was high [24]. Alsubaie et al. in Saudi Arabia reported that parents believed vaccines were unnecessary for rare diseases and that the number of vaccines received in each round was high. Guai et al. found that the biggest reason for vaccine hesitancy in Quebec was the belief that too many vaccines were given [29].

Wagner et al. found that 69% of mothers in India preferred that their children receive fewer vaccines at a time, 39% worried about vaccine side effects, 33% believed vaccines are no longer necessary for diseases that are no longer prevalent, and 20% believed new vaccines are more dangerous than old vaccines [22]. In general, most studies reported similar results. This could indicate the universality and extent of anti-vaccine actions and the spread of their beliefs through social media worldwide. The most common reason for COVID-19 vaccine hesitancy was the fear of possible complications in the fetus, according to a study conducted by Moini et al. (2023) [30]. According to Sherman et al. in England, parents refused the varicella vaccine due to concerns about side effects and not taking the disease seriously [31]. The present study was done during the COVID-19 pandemic, and 18.1% of mothers cited the fear of contracting Corona disease as a reason for not referring to a health center and the hesitancy of their child's vaccination. Parinyarux et al. in Thailand and Low et al. in Singapore also demonstrated that the Covid-19 pandemic increased parents' vaccine hesitancy in their children [32], [33]. Also, Sari et al. found that child vaccination coverage decreased during the COVID-19 pandemic in Abadan [34].

## Limitations

In this study, the questionnaire was completed according to the opinions of the child's mother. This is because, in the studied society, decisions about children's vaccinations are made mainly by mothers. However, in some cases, it may not reflect the family's opinions. Some mothers could not attend comprehensive health service centers or refused to participate for some reasons, including the COVID-19 pandemic, which forced them to fill out questionnaires via phone.

## Conclusion

According to this study, the biggest reasons for children's vaccine hesitancy were:

• The fact that the child was sick at the time of the injection
• The belief that vaccination is not needed to prevent uncommon diseases
• The worry about severe vaccine complications

Also, this study indicated the critical role of the mother's education, distance to the health center, and the type of vaccine in children's vaccination.

## Declaration of Competing Interest

The authors declare that they have no known competing financial interests or personal relationships that could have appeared to influence the work reported in this paper.

## Data Availability

Data will be made available on request.

## Footnotes

1 World Health Organization.

2 Content Validity Index.

3 Content Validity Ratio.
